# Preclinical models for the study of pediatric solid tumors: focus on bone sarcomas

**DOI:** 10.3389/fonc.2024.1388484

**Published:** 2024-07-18

**Authors:** D. Isabel Petrescu, Jason T. Yustein, Atreyi Dasgupta

**Affiliations:** ^1^ Aflac Cancer and Blood Disorders Center, Emory University, Atlanta, GA, United States; ^2^ The Faris D. Virani Ewing Sarcoma Center, Baylor College of Medicine, Texas Children’s Cancer and Hematology Centers, Houston, TX, United States

**Keywords:** preclinical models, cancer therapy, pediatric cancer, rare cancer, solid tumors, bone sarcomas, Ewing sarcoma, osteosarcoma

## Abstract

Sarcomas comprise between 10–15% of all pediatric malignancies. Osteosarcoma and Ewing sarcoma are the two most common pediatric bone tumors diagnosed in children and young adults. These tumors are commonly treated with surgery and/or radiation therapy and combination chemotherapy. However, there is a strong need for the development and utilization of targeted therapeutic methods to improve patient outcomes. Towards accomplishing this goal, pre-clinical models for these unique malignancies are of particular importance to design and test experimental therapeutic strategies prior to being introduced to patients due to their origination site and propensity to metastasize. Pre-clinical models offer several advantages for the study of pediatric sarcomas with unique benefits and shortcomings dependent on the type of model. This review addresses the types of pre-clinical models available for the study of pediatric solid tumors, with special attention to the bone sarcomas osteosarcoma and Ewing sarcoma.

## 1 Introduction

Cancer is the second most common cause of death in children aged 0–14 in the United States. In 2023, nearly 10,000 children from birth to 14 years of age and more than 5,000 adolescents from 15 to 19 years of age were expected to be diagnosed (1). Pediatric solid tumors are a subcategory that constitutes approximately 40% of pediatric cancers (2). Common types include brain tumors, neuroblastoma, Wilms tumors, germ cell tumors, rhabdomyosarcoma, Ewing sarcoma (EwS), and osteosarcoma (OS) (**Table 1**) (3). Although these are the most common types of pediatric solid tumors, these are still classified as pediatric rare cancers, as are all other types of pediatric cancer. Within the past decade, there has been a strong scientific focus on genes and genomics, including a renewed interest to determine how each individual’s body and their tissues and organs contribute a crucial role in the development of cancer. Efforts are now focusing on how to manipulate and exploit this special microenvironment to improve chemotherapy along with how to adapt tumor cells to be more susceptible to targeted inhibitors. There is a tremendous amount of evidence that it is not only the tumor cells, but also the host cells that can be reprogrammed to significantly contribute to tumor development and growth. Thus, it is imperative to study the tumor in its environment – both at the micro and macro scale. To do so, it is critical to employ model systems that can best mimic the natural, physiological state. These models not only help our understanding of tumor biology, but they also provide a platform for experimental therapeutic strategies. In this review, we address the existing and innovative preclinical models and their benefits and shortcomings for the study of these types of cancers with a focus on bone sarcomas.

**Table 1:** Characteristics of common pediatric solid tumors.

Disease	Incidence	Avg. age of onset	Clinical features	Genetic causes	Current treatment	Citations
Brain tumors	MB: Approx. 1 in 170,000 pHGG: 1 in 125,000	MB: 2–6 years of age pHGG: 0–14 years of age	Increased intracranial pressure	MB: chromosomal abnormalities, structural variants (differs for each of the 4 subgroups) pHGG: homozygous inactivation of p53 (*TP53*) and histone 3.3 (*H3F3A*), amplification of receptor tyrosine kinase *PDGFRA*	Surgery, combination chemotherapy (e.g., vincristine, cyclophosphamide, cisplatin, carboplatin, etoposide), radiation therapy	(3–7)
Neuroblastoma	Approx. 1 in 17,000	Between 18 and 22 months	Abdominal mass	*MYCN* amplification, chromosomal segment alterations (e.g., deletion at 1p, 11q, and gain at 17q), mutations in anaplastic lymphoma kinase (*ALK*)	Surgical resection, induction chemotherapy (cisplatin, cyclophosphamide, vincristine, doxorubicin, etoposide), radiation therapy	(3, 8, 9)
Wilms’ tumor	1 in 10,000	>80% of children diagnosed before the age of 5	Asymptomatic abdominal mass	Chromosomal abnormalities (e.g., gene deletions at 11p13 and 11p15), mutations in the *WT1* gene	Nephrectomy, chemotherapy (vincristine and dactinomycin), radiation therapy	(3, 10)
Bone sarcomas	EwS: Approx. 1 in 650,000 OS: Approx. 1 in 220,000 (children and young adults)	EwS: 10–19 years of age OS: 10–14 years of age	EwS: pain and swelling in affected bone OS: localized pain or swelling	EwS: chromosomal translocation between a member of FET family of RNA-binding proteins and a member of ETS family of TFs OS: aneuploidy, chromothripsis, kataegis, mutations in *TP53*	Induction chemotherapy (EwS: vincristine, doxorubicin, cyclophosphamide, ifosfamide, etoposide OS: doxorubicin, cisplatin or carboplatin, methotrexate), surgery	(3, 11–18)
Rhabdomyosarcoma	Approx. 1 in 230,000	Approx. 70% diagnosed before the age of 10	Soft tissue mass found in the extremities or head/neck	Alveolar RMS (ARMS): balanced chromosomal translocations between chromosome 1 or 2 and chromosome 13, *PAX3::FOXO1* and *PAX7::FOXO1* gene fusions Embryonic RMS (ERMS): chromosomal gains and losses, alterations of *RAS* family genes	Surgery, chemotherapy (vincristine, actinomycin D, cyclophosphamide), radiation therapy	(3, 19–22)

MB, medulloblastoma; pHGG, pediatric high-grade glioma; EwS, Ewing sarcoma; OS, osteosarcoma; TFs, transcription factors.

### 1.1 Pediatric solid tumors and need for preclinical models

A unique challenge with pediatric solid tumors is that symptoms can mimic common childhood illnesses, requiring careful attention from primary care providers to diagnose a solid tumor malignancy in children and adolescents (2). Unlike other solid tumors, the most common initially presenting symptom is pain or swelling for bone sarcomas (23). Furthermore, the current treatment strategy for most pediatric solid tumors remains limited to surgical resection, radiation therapy, and chemotherapy. Even with recent advancements in therapeutic options, there has been limited improvement in survival for most pediatric cancers, including solid malignancies such as metastatic sarcomas. This highlights the necessity to advance pre-clinical models of pediatric solid tumors for the purpose to identify new therapeutic targets and improve patient outcomes and survival.

An advantage of preclinical models is their utility as a method for testing new therapeutic targets. This is especially imperative for less common pediatric cancers for which specific therapeutic agents have yet to be identified (24). Due to the rarity of pediatric cancers, relatively low numbers of children are available as subjects for clinical trials assessing experimental therapies. For those that are available, their disease state is likely to be advanced or resistant to treatment (25). As an example, with the genomic complexity of OS, identifying therapeutic approaches that can be broadly applicable to most patients becomes challenging (11). Therefore, using complementary preclinical models is especially important to increase relevance of identified targets and therapeutic potential and to overcome the intrinsic disadvantages or challenges of each type of model when used in isolation.

### 1.2 Established models

**Figure 1:**
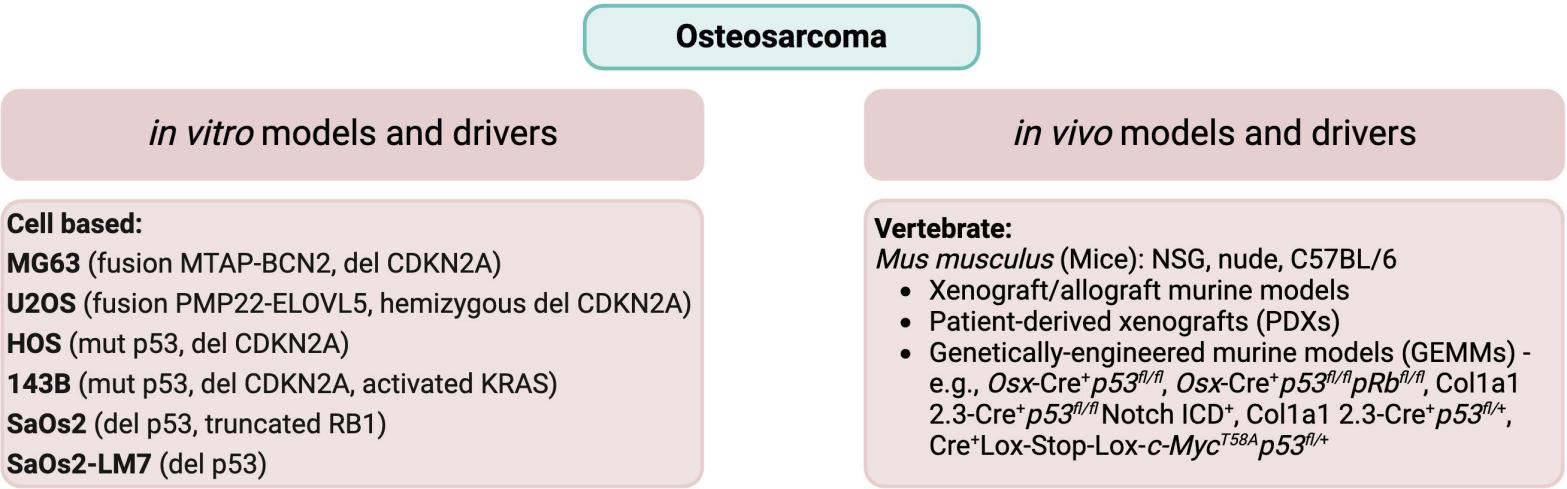
Common *in vitro* cell lines and *in vivo* animal models of osteosarcoma. Commonly used cell lines, including their genomic characteristics (26, 27), and *in vivo* animal models (28–30) are listed for osteosarcoma.

**Figure 2:**
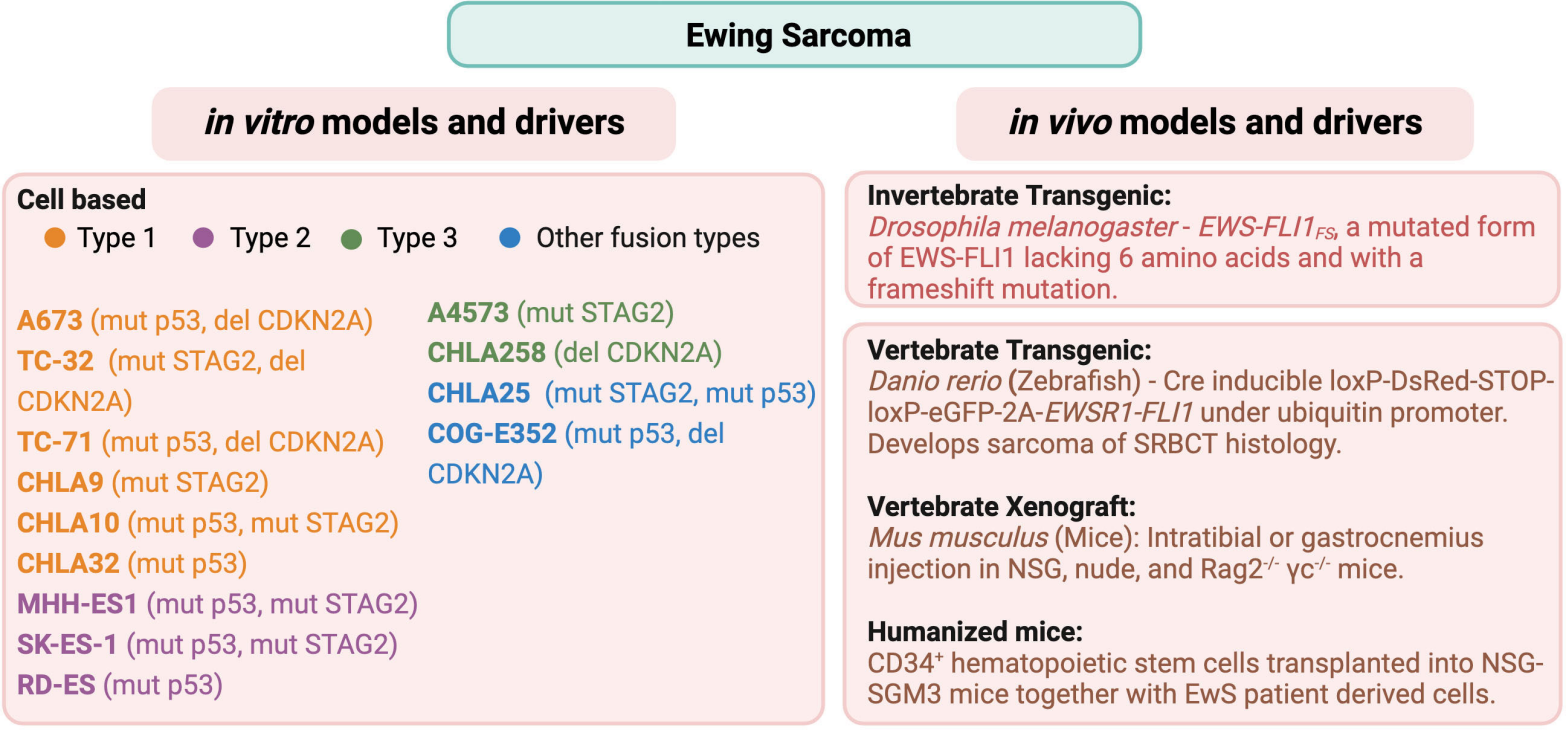
Common *in vitro* cell lines and *in vivo* animal models of Ewing sarcoma. Commonly used cell lines, including their fusion type (31) and genomic characteristics (32–36), and *in vivo* animal models (37–42) are listed for Ewing sarcoma.

Types of preclinical models can be broadly divided into the categories of *in vivo* and *in vitro*. *In vivo* models include animal models (spontaneous and xenografts), genetically engineered murine models (GEMMs), and patient-derived xenografts (PDXs). By contrast, *in vitro* models include cell culture, co-culture, organoids, and 3D model systems. *In vitro* models are similar in their function and use across most solid tumor types, whereas the various *in vivo* animal models can differ by type of sarcoma. For the purpose of this review, we will limit our focus to address *in vivo* models for the bone sarcomas osteosarcoma and Ewing sarcoma. A summary of the common *in vitro* (cell culture) and *in vivo* models of osteosarcoma and Ewing sarcoma is provided in **Figure 1** (OS) and **Figure 2** (EwS).

## 2 Osteosarcoma

OS is the most common primary malignant bone tumor, with bimodal peak incidence in adolescents and adults 60 years of age and older (43). The rate of childhood and adolescent OS is approximately 3 to 4.5 cases/million each year, with tumors most commonly developing in the lower long bones such as the femur, tibia, and fibula, as well as the humerus (44). OS tumors appear as a lytic intramedullary lesion that often invade and destroy the bony cortex and extend into nearby soft tissues. The major presenting sign is pain in the affected area (45). Approximately 20% of cases will present with metastatic disease, most commonly to the lungs. Metastases can also be found in additional bone, soft tissue, or lymph node lesions (12). The major treatment methods are surgery and chemotherapy. However, for OS patients with metastatic disease, the 5-year survival rate significantly decreases from approximately 70% to less than 40%, underscoring the need for novel therapeutic strategies (28). Patient-derived primary tumor cultures and genetically engineered murine models are frequently used pre-clinical models, which are complemented with available resources from the study of canine osteosarcoma, arising spontaneously in large breed dogs (11), to help gain insights into the molecular pathogenesis of the disease as well as assessing novel therapeutic regimens.

Studying canine osteosarcoma has proven to be valuable towards further understanding the molecular pathogenesis of OS and identification of potential therapeutic options for human osteosarcoma patients. There is evidence to suggest that transcriptomic and clinical patterns are comparable between canine and human OS. Of particular interest, the proportion of macrophage subtypes M0 and M2 present in the TME is similar between canine and human OS tumors (46). Furthermore, clinical trials of novel therapeutics are possible to be tested in canines before being approved for use in human patients (47). As an example, the drug auranofin (AF), an inhibitor of thioredoxin reductases (TrxRs), had been used in a clinical trial to evaluate its safety and efficacy in addition to the regular standard of care for the treatment of canine OS. The research findings demonstrated improved survival in male dogs when AF was combined with the standard of care treatment (48).

### 2.1 Molecular features

OS demonstrates genomic complexity without having characteristic features shared by every tumor. Nevertheless, there can be common features. Aneuploidy is a feature of many OS tumors. Furthermore, many tumors have been found to demonstrate chromosomal structural rearrangements, copy number gain, and copy number loss (45). Gene mutations in OS include *TP53*, which is the most frequently mutated, *RB1*, *ATRX*, and *DLG2* (11). Patients with Li-Fraumeni syndrome have a germline mutation in p53, resulting in an increased risk of developing OS (45). Similarly, patients with the inherited familial syndromes hereditary retinoblastoma, Rothmund-Thomson, Bloom or Warner syndrome have driver mutations for OS in *RB1*, *RECQL4*, *BLM*, and *WRN*, respectively (28). Chromosomal copy number gain is observed for sections of chromosomes 1p, 1q, 6p, 8q, and 17p. Conversely, copy number loss may include sections of chromosomes 3q, 6q, 9, 10, 13, 17p, and 18q. The oncogene *MYC* is found on chromosome 8q and has been found to be frequently amplified in OS (26). Several studies have also found associations with disease incidence at various loci, including single nucleotide polymorphisms (SNPs) within genes important for growth and development such as *IGF2R*, encoding the IGF2 receptor, *FGFR3*, encoding fibroblast growth receptor 3, and the genes *MDM2*, encoding the MDM2 p53 binding protein homolog with a role in the regulation of p53 function, and *TGFBR1*, encoding transforming growth factor-β receptor type 1 with a role in the regulation of cell proliferation (49). The dominant genomic drivers for several of the most commonly used OS cell lines are listed in **Figure 1**.

## 3 Ewing sarcoma

Ewing sarcoma (EwS) is a rare but aggressive bone cancer among children, adolescents, and young adults with an annual incidence rate in the United States of approximately 1 case per 1.5 million (50). Histologically, EwS appears as small round blue cells with a prominent nucleus, with tumors arising primarily in the pelvis and the proximal long bones: femur, tibia, and ribs (50). Other extraosseous sites include the thoracic wall, pleural spaces, and cervical and gluteal muscles (13). EwS cells commonly express CD99 on the plasma membrane and contain glycogen-rich cytoplasmic content (51). In radiological findings, EwS lesions appear as lytic, described as “moth eaten”, with frequent appearance of bone sclerosis, and newly formed, elevated periosteum appearing as lamellar (onion skin) or as appearance of displaced periosteum (Codman’s triangle) (50, 52).

With the advent of chemotherapy, the overall survival rate for children with localized EwS disease has increased to 70–75%. However, at least 25% of these patients will relapse, even after completion of the initial treatment plan. Another 25% of patients presenting with metastatic disease at the time of diagnosis have an abysmal 20–25% survival rate (53). This rate has not improved even after decades of research. Consequently, since the introduction of etoposide, no new therapeutic has been introduced to the standard treatment regimen (54). Thus, there is a serious need for novel approaches and therapeutic targets to be discovered.

### 3.1 Molecular features

Ewing sarcoma is characterized by the translocation of t(11;22)(q24;q12), resulting in the fusion of FET family of RNA binding proteins (EWSR1, FUS, and TAF15) to ETS (E-26 specific) family of proteins (FLI1, FEV, ERG, FEV, ETV1, ETV4, and ETV5) that act as transcription factors (55–57). The most common fusion protein, EWS::FLI1, arises from the fusion of Ewing sarcoma breakpoint region 1 protein (EWSR1) to Friend leukemia integration 1 transcription factor (FLI1) (31). EWS::FLI1 is the oncogenic driver for EwS in 85% of cases (13, 58). The EWS::FLI1 fusion protein is localized to the nucleus and binds DNA with the same sequence specificity as the parental FLI1 protein. Moreover, EWS-FLI1 acts as an efficient sequence-specific transcriptional activator, dependent on a transcriptional activation domain within the EWS sequence (59). Based upon the specific rearrangement of the EWS::FLI1 fusion protein, the type of EwS can be further classified. In more than 50% of cases, fusion of EWS exon 7 to FLI1 exon 6 (type 1) or to FLI1 exon 5 (type 2) is observed. The third most common fusion is between EWS exon 10 and FLI1 exon 5 (type 3). Lastly, fusion can be between EWS exon 10 and FLI1 exon 6 (type 4) (31). Based upon the genomic breakpoint, 12 types of EWS::FLI1 chimeric transcripts have been observed in patient samples (60).

EWS::FLI1 has been shown to bind to two different types of sites. The first site is at enhancers containing the ETS-2 binding motif, with a single GGAA as the core sequence. This is the binding site for the wild-type FLI1 protein. Compared to the FLI1 protein, the chimeric protein acts as a much more potent transcriptional activator, transforming NIH3T3 cells (61). The second site is specific to EWS-FLI1, consisting of microsatellite sequences of tandem GGAA repeats (GGAAμSats) (62), seen in many downstream target genes required for EwS tumorigenesis such as NROB1 (63). Transcriptional activity of EWS::FLI1 often leads to the formation of R loops made of 3 strands of DNA-RNA hybrid with single-stranded non-template DNA. Accumulation of R-loops can cause replication fork collapse, DNA damage, and increased activity in the Ataxia-telangiectasia mutated and Rad3-related (ATR) pathway (64). Indeed, due to these features, EwS demonstrates increased sensitivity to DNA damage repair inhibitors such as poly(ADP-ribose) polymerase (PARP) inhibitors. Thus, PARP and other DNA damage repair inhibitors are being investigated for targeted therapy in EwS (64, 65). Nonetheless, the genomic landscape of EwS is relatively stable with concomitant genetic alterations found only in a few genes such as mutations in TP53, STAG2, and deletion in CDKN2A as analyzed by several groups (32–36). We have mentioned the dominant genomic drivers for some of the most commonly referenced cell lines in **Figure 2**.

Microarray and chromatin immunoprecipitation analysis revealed EWS::FLI1 to be a direct or indirect transcriptional activator as well as a repressor for downstream targets (66, 67). Some of the direct activation targets are the neuronally expressed transcriptional repressor NKX2–2 (68); nuclear receptor NROB1, that plays a critical role in osteoblast differentiation and skeletal development (69); GLI1 (70), a member of the Sonic Hedgehog pathway; Id2, cMYC, CCND1 (71); EZH2 (72); and repression of the tumor suppressor FOXO1 (73). Other than direct transcriptional regulation, EWS::FLI1 contributes to sarcomagenesis by regulating alternative splicing (74, 75) and chromatin remodeling (76, 77).

Even though EWS::FLI1 hypothetically presents itself as a clear therapeutic target, a reliable, efficient inhibitor is still lacking. This remains a problem despite the detailed characterization of EWS::FLI1 as an aberrant transcription factor and a potent transforming gene (78–80). A major hindrance to developing a therapeutic inhibitor is the lack of EWS::FLI1-driven EwS murine models.

Ewing sarcoma appears to be a cancer specific to humans only. Many of the murine orthologs of EWS::FLI1 target genes lack GGAA binding sites (62). This, added with two other primary reasons perhaps explain why there remains a considerable challenge towards developing a reliable mouse model for Ewing sarcoma: 1) the lack of precision around its cell of origin; 2) although EWS::FLI1 appears to be the oncogenic driver, its overexpression results in cell death.

To design a model, one needs to have a definitive and rigorous understanding of the cell of origin, but for Ewing sarcoma it has been difficult to identify lineage-specific differentiation.

### 3.2 Cell of origin

Contrary to osteosarcoma, Ewing sarcoma lacks lineage-specific differentiation. The tumor is histologically classified as being formed from sheets of undifferentiated small round basophilic cells. Thus, the cell of origin of Ewing sarcoma is mired in controversy. It was first described by Dr. James Ewing in 1921 (81). Based upon cellular morphology, frequent presence of cells adjacent to vascular channels, and the presence of very little stroma, he described the undifferentiated tumor in the radius of a 14-year-old girl as “diffuse endothelioma of bone”, thus attributing an endothelial origin. Around the same time, a different school of thought identified similarities with neuroblastoma (82). Around 50 years later, based upon features resembling developing myelocytes, a myelogenous origin was suggested by Kadin and Bensch (82). One of the earlier theories suggested the origin was from primitive neuroectodermal cells, due to its histological resemblance to the tumor cells and expression of neuron-specific enolase (NSE) and S-100 markers in some EwS tumors (83–87). Gene expression patterns from ectopic expression of EWS::FLI1 in undifferentiated neural crest stem cells indicate similar patterns to that of EWS-FLI1 target genes (88). Hence the term primitive neuroectodermal tumors (PNETs) was used interchangeably with EwS. In 2013, WHO classified PNETs and EwS in one pathological entity under the umbrella term Ewing sarcoma family of tumors (ESFTs). WHO classification of tumors of soft tissue and bone in 2020 added a new category of “undifferentiated small round cell sarcomas of bone and soft tissue tumors” that now includes Ewing sarcoma and three new entities: *EWSR1*::non-*ETS* fusions, *CIC*-rearranged sarcoma, and sarcomas with *BCOR* genetic alterations along with prototypical Ewing sarcoma arising in bone or soft tissue (89). An alternative, more modern proposal for the cell of origin of EwS is the mesenchymal stem cell (90, 91). This is supported by the fact that knock-down of EWS::FLI1 in several different cell lines leads to a more distinct mesenchymal stem cell gene expression pattern (91). Additionally, EWS::FLI1 by itself can transform primary bone marrow derived MSCs and develop tumor with EwS features including small round cells, dependence on IGF-1, and expression of EWS::FLI1 target genes (92).

In attempts to generate a mouse model, three types of primary cell lines have been identified as permissive for EWS::FLI1 expression. These are mesenchymal stem cells (92, 93), neural crest stem cells (88), and embryonic osteochondrogenic progenitor cells (94, 95).

## 4 *In vitro* models

Types of *in vitro* models are comparable across various types of solid tumors, including the bone sarcomas osteosarcoma and Ewing sarcoma, highlighted in this review. *In vitro* models include two-dimensional (2D) cell culture, co-culture between tumor cells and cell types within the tumor microenvironment (TME), three-dimensional (3D) organoids/spheroids, and bioengineered 3D culture models.

### 4.1 Cell culture

Cell culture is widely used as a pre-clinical model across many cancer types, including for the study of pediatric solid tumors. A major advantage of cell culture is the relative ease of use, allowing novel therapeutic agents to be tested quickly and meticulously (96). Other advantages of cell culture are the use of standardized methods, the potential for rapid cell growth, and growth in translucent media, allowing cells to be monitored by light microscopy in real-time. In addition, cell culture is cost-effective does not require special equipment (97). The first cell lines for solid tumors were established in 1973 from tumor explants, with cell lines successfully developed for kidney, lung, and epidermoid carcinoma; rhabdomyosarcoma; glioblastoma and astrocytoma in the brain; and melanoma (98). In the decades since, the American Type Culture Collection (ATCC) (https://www.atcc.org/) has collected dozens of cell lines that are beneficial for the study of pediatric solid tumors. With the purpose to identify novel therapeutic targets, Shen et al. treated a panel of 19 well-characterized cell lines with more than 3800 unique chemical compounds (99). These cell lines were derived from solid tumors such as Ewing sarcoma, rhabdomyosarcoma, osteosarcoma, neuroblastoma, and central nervous system tumors (e.g., medulloblastoma, glioblastoma, atypical teratoid rhabdoid tumor). After completing dose-response curves for all compounds, a total of 62 compounds were found to be effective against 17 of the 19 cell lines. These classes of compounds included antineoplastic agents and immunosuppressants, antiparasitics, and anti-inflammatories, which had not been previously known to be effective against pediatric cancer cell lines. The antiparasitics mebendazole and difluoromethylornithine (DFMO) are being tested in clinical trials for pediatric brain tumors (NCT02644291) and neuroblastomas (NCT02395666) (99). Characterization of osteosarcoma cell lines could confirm differentiation capacity as well as tumor formation capacity *in vivo*, indicative of their utility as a strong representative model of the disease. However, a drawback of *in vitro* cell culture arises from the absence of stroma or the extra-cellular matrix. As a result, the microenvironment and tissue architecture of the tumor fails to be adequately represented, requiring other methods to supplement the cell culture model to study these features (100). A common *in vitro* method to study the interaction between tumor cells and immune cells within the tumor microenvironment (TME) is co-culture techniques (101). In this model, tumor cells are plated and grown in culture along with immune cell types.

### 4.2 Tumor microenvironment/co-culture

The multicellular co-culture model is considered advantageous for providing an approximate imitation of the communication network between the tumor and its microenvironment. A study into healthy human fibroblasts (HFs) co-cultured with the human osteosarcoma cell line MG-63 demonstrated morphological changes in the cell types compared to when HFs and MG-63 cells were grown independently. Furthermore, expression of biomarkers (i.e., YKL-40, VEGF, MMP1) was found to differ in the co-culture, with elevated expression of YKL-40 in HFs and VEGF in MG-63 (102). Possible methods for co-culture plating include direct or indirect mechanisms. With direct co-culture, cell types are layered on top of each other to allow for physical contact whereas with indirect co-culture, the different cell types are separated by porous membranes, such that non-contact-mediated signaling through secreted soluble factors can be investigated (101). For greater specificity and control of spatial arrangement, microfluidic techniques can be used to co-culture cancer cells with fibroblasts. As an example, tumor spheroids grown in co-culture with cancer fibroblasts in a microfluidic chip-based culture model were more resistant to the chemotherapeutic drug paclitaxel (103). Additionally, the tumor microenvironment can be investigated at sites of metastasis as well as the primary tumor. Mendoza et al. developed an *ex vivo* method called the pulmonary metastasis assay (PuMA) to study pulmonary metastasis, which is a common outcome of Ewing sarcoma and osteosarcoma disease progression. For this assay, GFP-expressing cancer cells proliferate in lung tissue with various other cell types present in the tissue culture such as migratory cells, type I and II pneumocytes, alveolar macrophages, vascular endothelial cells, fibroblasts, and red blood cells. This assay can be used to compare the metastatic ability of different human and murine cancer cell lines and to identify lung microenvironments conducive to metastasis (104). Taylor et al. investigated the role of EwS tumor cells in osteoclast formation and the osteolysis observed in EwS using a co-culture model system. EwS cell lines co-cultured with CD14^+^ monocytes primed with macrophage-colony stimulating factor (M-CSF) resulted in the formation of multinucleated cells positive for the osteoclast functional marker TRAP (tartrate-resistant acid phosphatase) (105, 106). Moreover, the co-culture of M-CSF-primed monocytes with EwS cell lines was able to cause lacunar resorption, a hallmark characteristic of osteoclasts. These results indicate that osteoclast-like cells are able to be formed from tumor-associated macrophages present within the EwS tissue cultures (106).

### 4.3 Organoids/spheroids

A recently developed *in vitro* model is three-dimensional (3D) culture systems to overcome challenges with 2D cell culture. The major advantage of 3D organoid/spheroid cultures is the potential to capture the complexity of the tissue environment in which a tumor resides (107). 3D spheroids can be formed by seeding a known density of cells into a 3D matrix scaffold, with the scaffold consisting of pores that are sufficiently small to restrict the size of the spheroids. The porous scaffold is supplemented with complete media then the plated cells are given time to grow into spheroids (108). Lawlor et al. developed spheroids of EwS cell lines TC32, A4574 and 5838 to investigate the roles of the RAS-RAF1-MEK-ERK 1/2 mitogen-activated protein kinase (MAPK) and phosphatidyl inositide-3-kinase (PI3K)-AKT pathways in the regulation of cell proliferation specifically regarding the regulation of cyclin D1 expression. Their results indicated that cyclin D1 protein expression required serum stimulation and cell-cell adhesion within the spheroid cultures grown in suspension. Moreover, the ERK1 and ERK2 kinases were found to be constitutively active in TC32 cells placed in suspension, suggestive of increased phosphorylation compared to monolayer cultures. Similar results were seen in 5838 and A4573 spheroids (109). He et al. described two distinct methods to establish OS organoids from primary tumors or lung metastases isolated from patients (110). The first method (“Cut/EnBloc”) used an inner transwell insert with a bottom layer of collagen gel matrix and minced tissue samples resuspended in collagen layered on top, with the transwell insert placed inside of a tissue culture dish. The second method (“Single-cell”) established each organoid from a single-cell suspension arising from the tissue samples. For this method, minced tissue samples were digested followed by treatment wth DNaseI and red blood cell lysis buffer. Lastly, cells were filtered through a cell strainer to recover single-cell suspensions, which were then embedded in Matrigel and allowed to solidify. The single-cell method was successful to establish organoids either from lung metastatic tissues or OS primary tumors. The authors concluded that the Cut/EnBloc method was more effective for establishing organoids from OS lung metastases whereas the single cell method was more successful for culturing organoids from primary OS tumor samples (110). A common function of 3D cell culture is to study the interaction between cells and the extracellular matrix (ECM). This can be particularly useful for testing drug efficiency by identifying whether the ECM prevents drug diffusion to cells (111). Benefits of spheroids are that their generation and use is simple and inexpensive. However, using spheroids to model tumor cells in 3D presents challenges for high-throughput screening, such as drug testing, and requires precise control of droplet size and size uniformity when developing the spheroids (112).

### 4.4 Engineered 3D culture

An emerging area of study in cancer research is the development of engineered *ex vivo* 3D cell culture models. This type of model is advantageous for its ability to recapitulate the shape and environment of the *in vivo* tumor. Each component of the TME can be experimentally modified to determine its effect on the tumor cells. Within 3D culture models, cancer cells, endothelial cells, and other stromal cells are co-cultured in a spatially relevant system representative of the native tumor environment. In this manner, hypoxia and the release of angiogenic factors in response to hypoxia can be examined and modified to mimic levels found in human tumors (97). To provide an example, cancer stem cells (CSCs) are a cell type present in cancer tissues that have the ability for self-renewal or differentiation into tumor cells. The 3D culture model is beneficial for understanding the interplay between CSCs and their niche in the tumor microenvironment (113). An innovative research study from Molina et al. involved engineering a 3D bone tumor niche using mesenchymal stem cells (MSCs) cultured on electrospun poly(ε-caprolactone) (PCL) scaffolds to imitate bone-like architecture, ECM, and mineralization with the goal to understand how components of the 3D environment would affect EwS cell growth, morphology, and signaling activation (114). Similarly, Bassi et al. designed two hydroxyapatite-based bone-mimicking scaffolds to focus on the CSC niche within the OS TME. The system consisted of 3D scaffolds together with enriched CSCs obtained by sarcosphere-forming culture originating from the OS cell lines MG-63 and SaOS2. The sarcospheres grown in the 3D scaffolds maintained their circular morphology, representative of the scaffold-free sarcospheres of the parental cells, while embedding within the scaffold matrix. Furthermore, quantitative PCR to analyze the expression profile of genes involved in stemness and niche communication between OS cells and CSCs revealed upregulation of the stemness genes NANOG and OCT-4 and the signaling gene IL-6 in the SaOS2 3D scaffold-based sarcosphere models in comparison to the scaffold-free sarcospheres. Likewise, the expression of NANOG and the signaling genes NOTCH-1 and HIF-1α was upregulated in the MG-63 3D scaffold-based sarcosphere models (115).

An exciting new area of research is the organ-on-chip model, also known as microfabrication. In this system, miniaturized organs or tissues are designed in a chip-like array, similar to a computer processor, that can be used for drug modeling. The advantages of the organ-on-chip model are its 3D design, modularity, versatility to represent different organ systems, and the possibility for rapid manufacturing of organ environments (116). Lu et al. designed an osteosarcoma-on-a-chip model by using a decellularized osteosarcoma extracellular matrix (dOsEM) that was loaded with extracellular vesicles from human bone marrow-derived stem cells (hBMSC-EVs) along with OS cells. These cell types were used as a bioink to create a micro-osteosarcoma (micro-OS) via 3D printing. The micro-OS was combined with a microfluidic system to establish an OS-on-a-chip (OOC) (117). After each of the multiple components was prepared and characterized, characterization of the OOC indicated that the OOC could mimic the OS bone marrow niche and exhibit high OS aggressiveness through activation of CXCL12/CXCR4 signaling to maintain PI3K/AKT-mediated proliferation and metastasis. Additionally, doxorubicin treatment of the OOC successfully simulated concentration changes of doxorubicin necessary to limit the viability of patient-derived OS cells *in vivo* (117).

## 5 Animal models

In OS, pre-clinical animal models are predominantly murine models. One specific advantage of murine models is that this type of model presents an invaluable method to study the metastatic cascade and to understand the sequential order of events for the development and metastatic spread of OS (14). Furthermore, various types of models can be combined in a study to address a hypothesis from multiple directions. As an example, Zhang et al. used a combination of *in-vitro* human OS cell lines along with three-dimensional (3D) culture models and tumor-bearing mice to identify the cyclin-dependent kinase 7 (CDK7) as a key player in OS growth and metastasis, resulting in the conclusion that targeting CDK7 or its target glucose-related protein 78-kDa (GRP78) presents a potential treatment strategy for OS (118).

In Ewing sarcoma, commonly used animal models are immunodeficient mice (*Mus musculus*). To a lesser extent, fruit flies (*Drosophila melanogaster*) and zebrafish (*Danio rerio*) may also be used as animal models to study Ewing sarcoma. Each of these models offers unique advantages and disadvantages. However, challenges remain with establishing animal models to accurately represent EwS tumors.

### 5.1 Xenograft/allograft murine models

In xenograft murine models, human cell lines are injected into immunodeficient mice, such as non-obese diabetic/severe combined immunodeficient (NOD.Cg-*Prkdc^scid^Il2rg^tm1Wjl^*/SzJ) (https://www.jax.org/strain/005557), commonly referred to as NSG or NOD *scid* gamma, NOD *Rag* gamma (NRG) (NOD.Cg-*Rag1^tm1Mom^ Il2rg^tm1Wjl^*/SzJ) (https://www.jax.org/strain/007799), and athymic immunosuppressed *nude* (NU/J) (https://www.jax.org/strain/002019) mice (119). Conversely, murine cell lines are injected into immunocompetent mice to establish allograft murine models (120). For this type of model, careful consideration for the site of injection is needed based on the experimental question. Each site of injection poses benefits and shortfalls for modeling OS tumor growth and progression (14). For example, injection subcutaneously (heterotopic) versus at the corresponding initial anatomical position for the tumor (orthotopic) affects the strength of the model to represent the complex process of carcinogenesis (121). Di Fiore et al. designed an OS model of CSCs by treating human OS MG-63 cells with 3-aminobenzamide (3-AB), a potent inhibitor of poly(ADP-ribose)-polymerase (PARP), which had resulted in massive cell death and the enrichment of a new CSC population, termed 3AB-OS. The 3AB-OS CSCs and MG-63 cells were injected into athymic nude mice to establish xenografts and determine the tumor-initiating capacity of each cell line. The results demonstrated that the 3AB-OS cells had the ability to form tumors *in vivo* whereas the MG-63 cells did not. Furthermore, the presence of Matrigel and the length of time for engraftment was demonstrated to affect tumor volume (122). In addition, for OS, the use of syngeneic murine cell lines in immunocompetent mice include MOS-J, K7M2, F420, K12, and POS-1 (37, 38, 123) provide an *in vivo* model with an intact, representative immune microenvironment (39),, which is not feasible when human OS cell lines are injected into immunodeficient mice.

Most EwS pre-clinical research depends upon developing xenograft tumors in immunocompromised mice. Although many researchers use subcutaneous, a more physiologically relevant orthotopic injection is intratibial or intrafemoral. For EwS cell lines that were originally extraosseous, gastrocnemius or intramuscular injections can be done. Several established EwS cell lines such as TC71, TC32, A673, RDES, SK-N-MC (124) are able to grow, develop, and metastasize in immunocompromised murine models. In these models, the primary site of metastasis is the lungs or secondary bone (125, 126).

Xenograft models allow for the characterization and targeting of tumor inherent properties. Thus, this type of model is suitable for pre-clinical drug testing, including bioavailability, for gene specific knock-in and knock-down effects, and for testing the efficacy of genetically-engineered chimeric antigen receptor T (CAR-T) cells. Visualizing the cell lines with luciferase or fluorescent proteins allows for live cell tracing and monitoring of metastatic spread. Using this method, we and several other research groups have shown distal metastasis typical for EwS in secondary bone, lungs, and liver. In addition, xenograft murine models were used to harvest organs and characterize *ex vivo* lung-specific metastasis in EwS (127, 128). Further, xenografts have been reported to be used to test and characterize cell lines that may be developed to be chemoresistant (129–131), radiation-adapted (132), or designed as organ-specific metastatic cell lines (128).

### 5.2 Patient-derived xenografts

**Figure 3:**
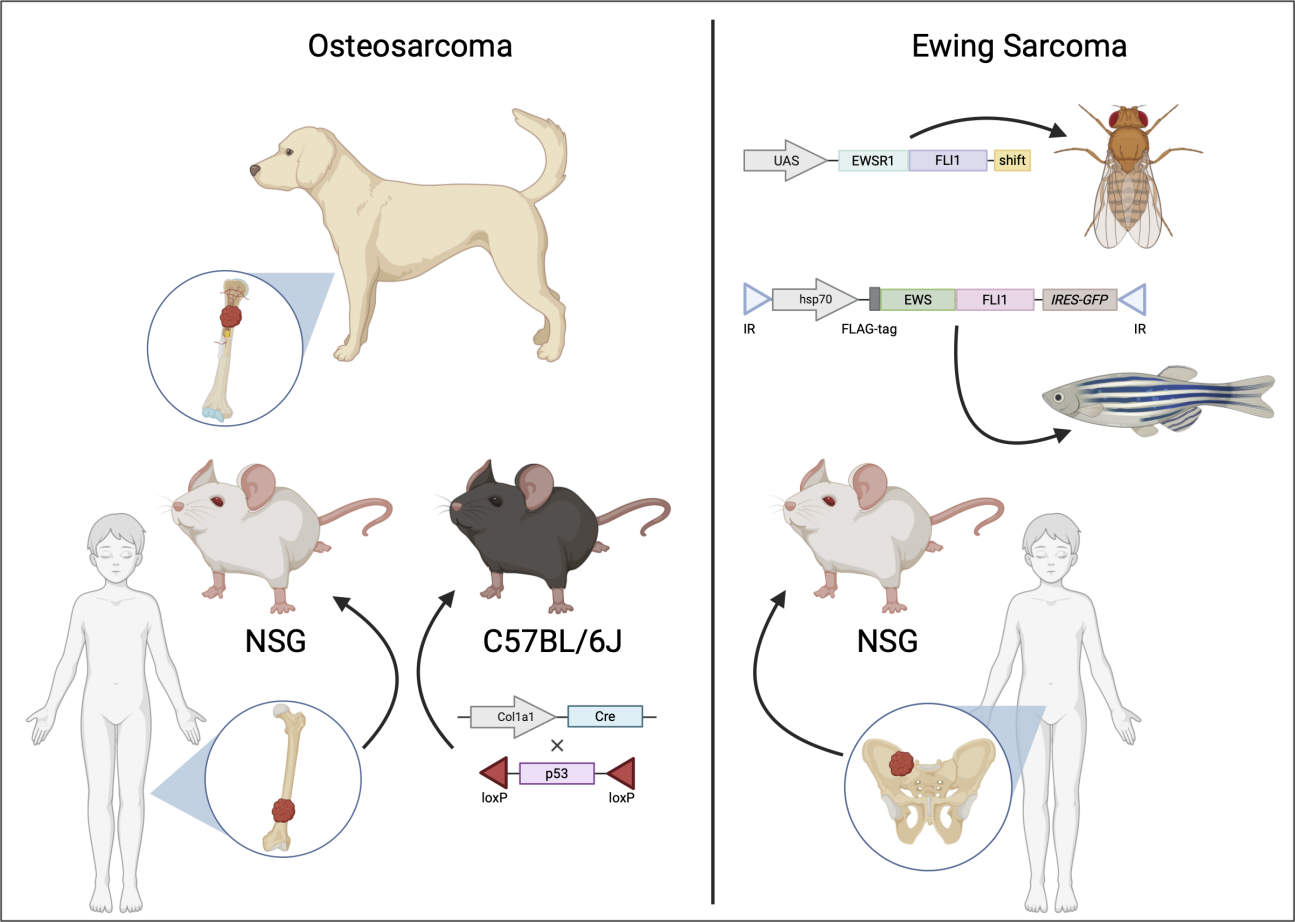
Animal models of osteosarcoma and Ewing sarcoma. Osteosarcoma (shown on the left) is often modeled using genetically modified murine models (GEMMs) [adapted from (135)] and patient-derived xenografts (PDXs) implanted in immunodeficient mice. Additionally, canine osteosarcoma closely resembles the human disease, providing insights into potential therapeutic options for human osteosarcoma. For Ewing sarcoma (shown on the right), *in vivo* pre-clinical models include PDXs or transgenic models such as *Drosophila melanogaster* [adapted from (42)] or zebrafish [adapted from (136)].

Patient-derived xenografts (PDXs) are advantageous for their ability to recreate the cellular, molecular, and histologic characteristics of the primary tumor. Furthermore, cell lines can be expanded from PDXs, with several possible uses, such as high throughput combinatorial drug screening (133). PDXs are established by transplanting biopsies from human tumors in immunodeficient mice (134) (**Figure 3**). However, success rates for the establishment of PDXs varies widely, depending on tumor type, tumor stage, mouse strain, and site of implantation.

Global efforts to develop PDXs for research and pre-clinical models include the EuroPDX consortium (www.europdx.eu/), the Public Repository of Xenografts (www.proxe.org), and the Patient-Derived Models Repository (www.pdmr.cancer.gov/) from the National Cancer Institute (NCI) (137). Specific to pediatric solid tumors, St. Jude Children’s Research Hospital has established the Childhood Solid Tumor Network (CSTN) repository of PDXs (www.cstn.stjude.cloud/). Another available repository is the Baylor College of Medicine PDX Portal (www.pdxportal.research.bcm.edu/pdxportal/).

PDXs were used as *in vivo* models to measure the drug response to vincristine or cyclophosphamide in pediatric solid tumors, including Wilms tumors, sarcomas, brain tumors, and rhabdoid tumors. The results of this study indicated broad-spectrum activity of vincristine and cyclophosphamide across cancer types, providing encouraging evidence to use *in vivo* PDX models for the identification and validation of novel therapeutics (25). With regards to the study of OS, many groups have developed orthotopic OS models, termed patient-derived orthotopic xenograft (PDOX) models, so that the transplanted tissue can grow at a location that most closely resembles the site of spontaneous disease in patients (26, 138). One of the disadvantages of the PDX model is the inherent inability to study immune system interactions and the tumor microenvironment due to the requirement of immunodeficient mice in order to establish the model (139). Although PDXs in immunocompromised mice lack the developmental pathway and the systemic environment of a transgenic model, compared to cell-derived xenografts (CDXs), they are better able to retain crucial tumor microenvironment, which has a significant role in tumor growth and development, partially due to tumor cell infiltration and the stromal component of the tumor tissue. Indeed, when compared to clinical patient samples, gene expression profiling from different PDX models shows a closer similarity than CDXs (140). This result remained consistent over several passages in mice (141, 142). PDXs can circumvent issues seen with low clinical correlation when chemotherapy agents are tested on cell-derived models (143). In addition, orthotopic transplantation of PDXs followed by hindlimb amputation provides a method to mimic surgical resection in OS patients and to allow time for the development of spontaneous metastases, such that therapies can be identified to target distant metastases (144).

Using mostly NSG or nude mice (5–10 weeks old), several groups (137, 140–142, 145, 146) have generated Ewing sarcoma PDXs from both local as well as metastatic samples using incisional or percutaneous biopsies in patients with varying success rate of engraftment of 25–45% (137, 141, 145). The rate of engraftment was positively correlated with relapse in newly diagnosed EwS patients (145).

Drawbacks to this type of model include the fact that over time, tumor infiltrates from patient samples can be replaced by host immune and stromal cells resulting in differential gene expression patterns between the primary patient samples and serial implants in mice. With successive implantation in mice, there is a high chance of clonal selection to occur, with expansion of the most aggressive subpopulations to dominate the tumor cell population. Additionally, there can be cross-species signaling mismatch between signaling molecules of the host and the human receptors present in the tumor (140, 145).

### 5.3 Transgenic models

For transgenic models of OS, the most commonly used model is genetically engineered murine models (GEMMs) (**Figure 3**). Due to the fact that GEMMs are not easily feasible for EwS, available transgenic models are the fruit fly (*Drosophila melanogaster*) and the zebrafish (*Danio rerio*) (**Figures 2**, **3**).

#### 5.3.1 Genetically engineered murine models

Genetically engineered murine models (GEMMs) (**Figure 3**) are a common method to study tumor biology for several types of cancer, including OS. Advantages of murine models include the small size of mice, that mice reproduce quickly and produce large litters, and with technical advancements, including CRISPR/Cas9, mice can be genetically manipulated with relative ease. Types of mouse models can be grouped into various categories, depending on the intended purpose of the model. These categories include transgenic and gene-targeting approaches for either loss-of-function or gain of function studies (147). Although there are numerous advantages, a disadvantage of GEMMs is that they present a major commitment in the cost, effort, and time required to develop and maintain these models (139).

Specifically in the context of OS, one of the benefits of GEMMs is their potential use for gaining further insights into genetic and molecular mechanisms of OS development, such as by identifying driver mutations and/or mutations that co-exist in both normal and tumor cells (28). As an example, a murine model using conditional (floxed) alleles of both *p53* and *pRb* or *p53* alone to allow for tissue-restricted gene inactivation of osteoblasts following Cre expression resulted in the development of murine OS, indicating the requirement for disruption of *p53* (29). Another study using GEMMs had identified a role for Wnt signaling in the development of OS through the activity of c-Fos and Loxl2. c-Fos, a member of the Activator Protein-1 (AP-1) transcription factor complex binds to the promoter of each of the Wnt ligands *Wnt7b* and *Wnt9a*, which is followed by expression of the collagen-modifying enzyme Loxl2 dependent on the transcription factors Zeb1 and Zeb2. c-Fos GEMMs treated with the pan-Lox inhibitor BAPN or specific Loxl2 blocking antibodies exhibit delayed OS development (148). In like manner, osteosarcoma-specific GEMMs were established utilizing an osteoblast-specific Cre allele crossed with floxed *p53* or Lox-Stop-Lox (LSL) R172H-mutated *p53* alleles. These GEMMs represent a closely representative model of human OS tumor development and progression. Microarray analysis of both localized and metastatic tumors developed from these models revealed downregulation of the naked cuticle homolog 2 (NKD2) gene, a negative regulator of Wnt signaling, that was found to be important for the suppression of OS tumor growth and metastasis (135). In addition, approximately 20–30% of OS tumors exhibit amplification of chromosome 8q24, the chromosomal location of the oncogene *c-Myc*, which correlates with a poor prognosis (30, 149, 150). Recently, an osteoblast-specific Myc knock-in OS model was generated and molecularly characterized by Nirala et al. (30). This model leads to acceleration of tumor development, with a high incidence of metastasis and homologous gene signatures seen in human tumors (30).

Ewing sarcoma is a tumor occurring only in humans, and there are no records of EwS in mice or any other species (13, 95, 151). Thus far there have been no reliable, viable murine models that recapitulate Ewing sarcoma development and progression. Several attempts at generating murine models in recent decades have either caused embryonic lethality, developmental defects, or failed to create any reliable genetically engineered mouse model for EwS that could consistently reciprocate the histological, epigenetic, or proteomic features attributed to EwS (152). The lack of a clear lineage-specific promoter confounds the issue further, even though the uniquely expressed translocated protein EWS::FLI1 is the oncogenic driver. A second set of problems arises from the fact that the cis-regulatory enhancers with GGAA microsatellites, the binding site for EWS::FLI1, are poorly conserved (13). Transgenic expression of EWS::FLI1 under a Prx1 promoter – a promoter that is activated in undifferentiated mesenchyme transitioning to osteoblasts during early differentiation in the developing limbs (40, 153), resulted in abnormal development with no tumor formation (41). Using Osterix (osteoclast-specific) or Mx1-*Cre* conditional models, development of erythroid or myeloid leukemias were observed, but development of sarcomas failed to occur (95, 154). Attempts with other promoters targeting EWS::FLI1 or *Cre* expression include Runx2, Col1a2.3, Col1a3.6, CAG, Nse, NEFL, Dermo1, P0, and Sox9 (95). Together, it would appear the co-occurrence of several factors that are yet to be discovered, including the cell of origin, the development phase, promoter leakiness, other co-factors, binding sites, other oncogenic molecules are critical for Ewing sarcoma to develop.

#### 5.3.2 Drosophila model

Although development of a realistic model is not physiologically or anatomically permissive in *Drosophila*, there is a strong conservation of molecular pathways with humans. Thus, there is potential in using a *Drosophila* model to reproduce EWS::FLI1 regulated oncogenic pathways (**Figure 3**). A recent report used a spontaneous mutant variant – EWS::FLI_1FS_ to create transgenic lines in *Drosophila* (42). EWS::FLI_1FS_ is a truncated protein with six amino acid loss with a C-terminal frameshift retaining the entire EWSR1 and most of the FLI1 including the DNA binding domain. Using this construct, the group could circumvent EWS::FLI1-mediated cellular toxicity and induction of apoptosis. Proteogenomic analyses revealed EWS::FLI_1FS_ interacts with known homologues of EWS::FLI1 interacting proteins, including transcription, chromatin remodeling, and spliceosome factors. This model could also demonstrate activation of transcription from the two types of EWS::FLI1 binding sites.

#### 5.3.3 Zebrafish

Zebrafish (*Danio rerio*) has approximately 70% overlap with the human genome (155). They are almost transparent; hence, internal development can be easily observed. Hundreds of embryos that develop outside the mother’s body can be studied for a limited cost. Thus, they provide an attractive model to trace early-stage tumor development. In one study from Leacock et al., in p53-deficient embryos, transposon mediated mosaic expression of EWS::FLI1 (**Figure 3**) developed into peripheral nerve sheath tumors and in rare numbers, into leukemia-like or small round blue cell tumors (SRBCT), partly resembling EwS in histological features and gene expression patterns. The limitations of this model were low penetrance and the co-occurrence of other tumors (136). In a more recent study, a zebrafish model based upon Cre-inducible expression in a wild-type background reported high penetrance with rapid onset of SRBCTs. The EWS::FLI1-expressing tumors were positive for EwS markers such as CD99 and transcriptional targets such as activation of MAPK/ERK pathway (156).

### 5.4 Humanized murine models

In the absence of GEMMs for EwS, attempts have been made by several research groups to use humanized murine models, particularly in the context of studying tumor immunotherapy and the TME of EwS (157). In these studies, fresh human cord blood CD34+ hematopoietic stem cells were intratibially injected into 3–4 weeks old NSG-SGM3 mice along with EwS patient-derived cells. Reconstitution of T cells, B cells, natural killer (NK) cells and monocytes could be observed (157). The main caveats of humanized mouse models are the cost-prohibitory aspect along with the technical and regulatory challenges they can pose. One important disadvantage of this model includes the species difference between each cytokine and its corresponding receptor, resulting in a significantly reduced level of myeloid and NK cells due to the lack of crosstalk (158). Humanized mice have a reduced life span and can develop anemia and other pathological conditions due to an imperfect immune system (157, 159). Further advancements in this field and subsequent utilization of these models are an active area of research.

## 6 Applicability

The benefits of preclinical models can be directly measurable by considering that therapeutics tested using preclinical models have been approved for clinical trials and translated into the clinical setting for the treatment of patients with pediatric solid tumors. One such small molecule is cabozantinib (CBZ) in a phase II clinical trial (NCT02243605) completed for patients with relapsed EwS or OS. The cabozantinib in patients with advanced Ewing sarcoma and osteosarcoma (CABONE) phase II clinical trial, conducted as a collaboration between the National Cancer Institute (NCI) and the French Sarcoma Group, recruited patients from ten centers in the French Sarcoma Group, with 45 patients for each sarcoma. Most patients with EwS and 50% of OS patients treated with cabozantinib exhibited tumor shrinkage (160). CBZ is a tyrosine kinase inhibitor that targets multiple tyrosine kinase receptors, including vascular endothelial growth factor 2 (VEGF2), MET, and rearranged during transfection (RET) proto-oncogene (161, 162). Given that MET has been found to be overexpressed in OS, Fioramonti et al. evaluated the effect of CBZ on OS using *in vitro* OS cell line models or co-culture models of OS cells with bone cells to reproduce the tumor microenvironment interactions. Their results indicated that CBZ treatment could inhibit OS cell proliferation in a co-culture model system through a mechanism dependent on the expression of receptor activator of nuclear factor κB (RANK) by the OS cells. CBZ treatment had also inhibited OS proliferation and cell migration, an indicator of invasion and metastasis in tumor cells, when OS cells were cultured independently (161).

In Ewing sarcoma, attempts to directly target the tumor-specific EWS::FLI1 protein using the inhibitor TK216 in patients with recurrent/refractory EwS (NCT05046314) is now in a phase II clinical trial. TK216 is an analog of YK-4–279, an inhibitor that blocks RNA helicase A binding to EWS::FLI1, which had been identified from a drug screen. TK216 was tested in pre-clinical models to induce apoptosis in EwS cells and to inhibit tumor growth in murine xenograft models (163). From pre-clinical studies, insulin-like growth factor-1 receptor (IGF1R) was found to be an important therapeutic target in EwS (164). Although several phase I trials showed moderate effect in a small number of patients, successfully transitioning to phase II trials include the combination of IGF1R inhibitor (cixutumumab) and an mTOR inhibitor (temsirolimus) for patients with refractory EwS (NCT01614795). This trial showed a promising response rate of 29% (165). Cell line models have demonstrated a significant association between presence of EWS-FLI1 and sensitivity to PARP inhibitors such as olaparib (65). However, xenograft models identified the limitation of olaparib as a single agent while highlighting its efficacy in combination with other agents (166, 167). Similarly, clinical trials have not shown significant efficacy for the PARP inhibitors olaparib or talazoparib as single agents (NCT01583543, NCT02116777) and demonstrated moderate success in combination with irinotecan alone or with temozolomide (NCT04901702).

Many other chemotherapeutics currently in clinical trials have been previously tested and validated using pre-clinical models. Nonetheless, there are many chemotherapeutics that fail to transition from pre-clinical models into clinical trials beyond phase I. As such, there is a special importance for pre-clinical models to closely resemble the spontaneous disease in order for chemotherapeutics and targeted agents to be strong candidates for treatment options before being tested in clinical trials and introduced to patients. With this in mind, it is especially important to account for pharmacokinetically clinically equivalent drug doses with treatment of human tumor xenografts such that similar results can be observed when testing the drugs in a clinical setting (168). Studies making use of multiple types of pre-clinical models are promising so that shortfalls with one type of model can be overcome with the use of complementary models.

## 7 Discussion

Over the past couple of decades there has been an explosion in advancements in genomic analysis of patient tumors and identification of genetic mutations or alterations that can be candidate therapeutic targets. While these tremendous advancements in profiling patient tumors have allowed for the development of personalized medicine and a greater understanding into cancer biology, the validation of these genetic aberrations as therapeutic targets relies heavily on the development and applications of preclinical models that represent the molecular, cellular, and biophysical landscapes of the specific tumor types.

As we have outlined and described above for pediatric bone sarcomas, there has been the development and characterization of numerous *in vitro* and *in vivo* preclinical models ranging from direct patient tumor xenografts and cell lines to 3D organoid and microfluidic platforms to genetically engineered murine, zebrafish, and even *Drosophila* models. The establishment of these models has not only allowed further molecular profiling of patient tumors, but also the development of complementary, cross species comparative oncology studies to further identify and define key genomic features of individual cancer types that are candidate therapeutic targets and models that can be used for preclinical testing.

However, the field is still actively working towards improving these approaches in model development to enhance their representation of both tumor *intrinsic* and *extrinsic* (e.g., tumor immune microenvironment, cancer-associated fibroblasts, etc.) patient tumor biology, at different stages of their development, which will allow for more efficient and accurate identification of effective therapies. As it has been an exciting era in molecular biology and tumor genomics, it has been an equally stimulating time in biomedical engineering and preclinical animal models, with innovative technical approaches that have improved our ability to accurately model both tumor intrinsic and extrinsic tumor development and evolution. Applying the armamentarium of spatial and single cell multi-omics analysis to these models has already increased our insights into patient tumor biology. Further advancements in technology and integration of preclinical models will undoubtedly improve our ability to provide real time patient tumor profiling and testing of individualized therapies that will lead to improved outcomes for those conditions in dire need of improved therapeutic interventions.

## Author contributions

DIP: Conceptualization, Visualization, Writing – original draft, Writing – review & editing, Investigation. JY: Conceptualization, Funding acquisition, Resources, Supervision, Writing – original draft, Writing – review & editing. AD: Conceptualization, Funding acquisition, Visualization, Writing – original draft, Writing – review & editing, Investigation, Supervision.
